# Racial discrimination and anti‐racist action: The mediating effects of fair‐society belief among Filipino American and Korean American young adults

**DOI:** 10.1111/jora.70023

**Published:** 2025-05-02

**Authors:** Michael Park, Bongki Woo, Eunseok Jeong, Bryan Gu, Yoonsun Choi, David Takeuchi

**Affiliations:** ^1^ School of Social Work Rutgers University New Brunswick New Jersey USA; ^2^ College of Social Work University of South Carolina Columbia South Carolina USA; ^3^ Crown Family School of Social Work, Policy and Practice The University of Chicago Chicago Illinois USA; ^4^ School of Social Work University of Washington Seattle Washington USA

**Keywords:** anti‐racist action, fair‐society belief, racial discrimination

## Abstract

Racial discrimination is an important developmental context for racially minoritized young adults, contributing to the formation of societal beliefs and civic commitments. While discrimination is understood to motivate anti‐racist action in young adults, the contributions of young adults' belief in a fair society toward this relationship remain unclear. This study examined fair‐society belief as a mediating mechanism through which Asian American young adults interpret experiences of discrimination and translate them into various anti‐racist actions. Using data from Filipino American (*n* = 265, *M*_age_ = 22) and Korean American (*n* = 347, *M*_age_ = 21) young adults during the COVID‐19 pandemic in 2022, we conducted path analyses to test the mediating effects of fair‐society belief in the relationships between COVID‐19‐specific and general discrimination and anti‐racist action. Discrimination was found to motivate Asian Americans to engage in anti‐racist actions, although fair‐society belief served as a significant mediating factor only for Filipino Americans, particularly those who are U.S.‐born, in the link between COVID‐19‐specific discrimination and interpersonal anti‐racist action. These findings suggest heterogeneity in Asian Americans' response to discrimination and underscore the importance of considering specific ethnic contexts when developing programs to promote anti‐racist action in Asian American young adults.

## INTRODUCTION

Racial discrimination jeopardizes the health and well‐being of those targeted (Gee et al., 2009; Ho & Çabuk, 2023). Although anti‐Asian discrimination is not a new phenomenon, the COVID‐19 pandemic led to a dramatic surge in anti‐Asian discrimination, violence and hate, which posed significant challenges for Asian Americans (Cheah et al., 2020; Darling‐Hammond et al., 2020; Yellow Horse et al., 2022). Theoretical (Watts et al., 2003) and empirical literature (Christophe et al., 2022; Hope et al., 2022; Park et al., 2024; Zhang et al., 2022) suggest that experiences of racial discrimination may motivate anti‐racist and other forms of civic action among targeted groups and individuals. However, the mechanisms underlying this relationship remain underexplored. For Asian American young adults, along with other racially minoritized groups, it is possible that racial discrimination may heighten their awareness of social and racial inequities and subsequently inspire civic action (Wong et al., 2011). To fill this gap, the present study examined fair‐society belief as a mediating mechanism through which Asian American young adults interpret experiences of discrimination and translate them into various anti‐racist actions.

### Asian American anti‐racist action

Racism is a significant developmental context for racially minoritized people (Choi et al., 2020; Coll et al., 1996; Hope et al., 2015; Iwamoto et al., 2022; Seaton et al., 2018), and anti‐racist activism may serve as an outlet for the discriminated to both cope and demonstrate resilience (Berman & Paradies, 2010; Hope et al., 2022; Mattis et al., 2002; White‐Johnson, 2012). As racism is multifaceted, anti‐racist action can also follow a complex pattern. Anti‐racist action seeks to confront racial bias, remove racial inequities, and advance racial justice through individual and collective efforts (Aldana et al., 2019). Specifically, Aldana et al. (2019) operationalized anti‐racist action into three levels: interpersonal action (e.g., defending the target of racial slurs), communal action (e.g., joining a club or organization working on issues related to race), and political change action (e.g., engaging in protests). This study utilized this conceptualization to investigate the predictors of different forms of anti‐racist action among Asian American young adults.

A greater focus was given toward Asian American anti‐racist action during the particularly discriminatory climate of the COVID‐19 pandemic. Asian American organizations demonstrated community action by fundraising, organizing resources, and providing bystander trainings (Zhou, 2022). Those who personally experienced racial discrimination participated more in anti‐racist actions (Jun et al., 2021), and critical reflection on systemic inequities predicted greater support for anti‐racist causes, including movements like Black Lives Matter, which advocated for other racial minority groups (Matriano et al., 2021; Saavedra & Yoo, 2023). These study findings suggest that exposure to systemic inequalities may influence anti‐racist action, but it remains unclear whether a belief (or lack thereof) in the fairness of society is linked to the relationship between discrimination and anti‐racist action.

### Discrimination and anti‐racist action

There are at least two divergent possibilities in how young Asian Americans respond to discrimination; that is, it can either encourage anti‐racist activism or lead young adults to withdraw from it. The Sociopolitical Development Theory (Watts et al., 2003) argues that racial discrimination can strengthen understanding of racial injustice and encourage civic action from the affected individuals (Hope & Jagers, 2014). For example, perceptions of inequity or experiences of discrimination increased involvement in civic activities among Asian Americans (Lui et al., 2022; Park et al., 2024; Wong et al., 2011). However, experiencing discrimination may also make individuals feel excluded from society, believe less in civic institutions, and alienate them from activism (Rumbaut, 2008; Sánchez‐Jankowski, 2002). Although discrimination has been shown to be related to civic perceptions (e.g., civic beliefs and civic satisfaction), other studies do not find discrimination related to civic actions (e.g., protesting, expressing political opinions; Ballard, 2015; Cooc & Kim, 2021). These divergent findings indicate a need to examine potential mediating factors, such as fair‐society belief, that may help explain the relationship between discrimination and civic engagement.

### Fair‐society belief as a mediator

Fair‐society belief refers to the notion that society is fair, with equal opportunities and impartial treatment by social institutions for all (Flanagan, Syvertsen, & Stout, 2007). However, minoritized individuals, who often experience structural and personal discrimination, may find it hard to maintain this belief. Although youth, regardless of ethnic background, have been found equally likely to endorse the belief that the United States and its associated social structures are fair (Flanagan, Cumsille, et al., 2007), youth who experience or perceive discrimination are less likely to endorse those beliefs (Ballard, 2015; Flanagan et al., 2009; Wray‐Lake et al., 2008). In a study of American adolescents of African‐, Arab‐, Latinx‐, and European‐American backgrounds, adolescents' experiences of discrimination were negatively associated with adolescents' belief that America was a fair society (Flanagan et al., 2009). A qualitative study of Latinx adolescents' response to immigration policy during the Trump administration found that adolescents' criticisms of the administration consistently included a failure to uphold a tenet of fairness (Wray‐Lake et al., 2018). Experiences of discrimination decrease the belief that society is fair.

Extant literature has identified belief in a fair society as a critical determinant of civic and political involvement. This claim is theoretically supported by the Sociopolitical Development Theory (Watts et al., 2003) and the Critical Consciousness Theory (Watts et al., 2011), both of which recognize the importance of social perspectives and analysis as central features of political development for young adults. However, empirical studies examining the relationship between youths' civic perspectives and resultant action suggest ambivalence with regard to fair‐society belief's influence in determining civic action. On the one hand, empirical studies find that belief in society's fairness positively predicts personal civic commitments (Flanagan, Cumsille, et al., 2007). On the other hand, perceptions of society being unfair may motivate more action to restore fairness. For example, although not directly indicative of fair society belief, research suggests that critical reflection of structural racism, in fact, promote anti‐racist action (Bañales et al., 2020; Diemer & Rapa, 2016; Golden & Byrd, 2022; Watts et al., 2011), suggesting that recognition of society's unfairness may motivate critical action.

The seemingly inconsistent findings in prior research can be partly explained by cultural and ethnic differences, as recent studies have shown that the effects of fair‐society belief on civic engagement are heterogeneous and culturally dependent. For example, in a study of Asian American and Latinx American adolescents, Wray‐Lake et al. (2015) found that the relationship between fair‐society belief and civic outcome differs by ethnicity. Fair‐society belief was both higher and positively predicted civic commitments in Asian adolescents, while Latinx adolescents reported lower fair‐society belief and showed no predictive relationship with civic commitments. The authors posit that this difference reflects the distinction between each group's social positioning: the Asian group's status as a “model minority” contrasted with the more exclusionary position of the Latinx group (Junn, 2007). These findings reflect important ethnicity‐based differences in fair‐society belief. Understandably, ethnic/racial groups characterized by inclusion have been found to view American society more positively (Deaux et al., 2006) and are more likely to engage in society to reciprocate positive experiences (Sánchez‐Jankowski, 2002). Thus, while fair‐society belief can largely be understood to encourage civic action, it is important to recognize the differential impact such belief can have within different social contexts.

The mixed findings may also stem from existing studies' failure to account for crucial social positions such as nativity status. For example, first‐generation immigrants may have come from societies with different contexts—such as socio‐political and/or economic—which can shape how they perceive fairness in the United States (Kao & Tienda, 2022). For example, studies suggest that experience in home countries influence immigrants' attitudes toward crimes and authorities, such as the police (Correia, 2010; Menjívar & Bejarano, 2004). It is also possible that individuals who chose to migrate—often driven by hopeful ideals about opportunity and a better future (Kao & Tienda, 2022; Perreira et al., 2006)—may be more inclined to believe that society is fair. Meanwhile, second‐generation immigrants may have a greater awareness of inequity and, subsequently, less favorable views of the United States (Jensen, 2010; Perreira et al., 2006), which may result in lower endorsement of similar beliefs. This claim is supported by empirical research, which found that first‐generation immigrant youth were more likely to endorse fair‐society beliefs than their second‐generation counterparts (Wray‐Lake et al., 2015). Moreover, extant research indicates that the impact of racial discrimination on the development of racially minoritized youth is more prominent among the U.S.‐born than the foreign‐born (Armenta et al., 2013). These findings suggest that nativity status may significantly moderate how fair‐society beliefs mediate the relationship between discrimination experiences and anti‐racist actions. However, this potential moderating effect remains unexplored in the current literature.

### The present study

We used a sample of Filipino American and Korean American young adults to examine whether and how fair‐society belief mediates the relationships between racial discrimination and anti‐racism actions. We hypothesized that fair‐society beliefs would significantly mediate the relationships between experiences of COVID‐19‐related and general racial discrimination and anti‐racist actions. Specifically, we hypothesized that experiences of COVID‐19‐related and general racial discrimination would be associated with less fair‐society beliefs, which in turn would be associated with more anti‐racist actions. Additionally, we tested whether these relationships further vary by nativity (U.S.‐born, foreign‐born) within each ethnic group. We expect that the mediating relationships, if present, would be more salient among the U.S.‐born than the foreign‐born groups.

## METHODS

### Data

This study analyzed the data collected from the fourth wave of the Midwest Longitudinal Study of Asian American Families (MLSAAF) project that has been following Filipino American and Korean American families in the Midwestern U.S. since 2014. The Wave 4 data was collected during the COVID‐19 pandemic from April 2021 to April 2022. In total, 612 Asian American young adults (18–22 years old) participated in the survey, including 265 Filipino Americans and 347 Korean Americans at Wave 4. The average age was 21.5 for Filipino Americans and 21.2 for Korean Americans. About 70% of Filipino Americans and 60% of Korean Americans were U.S.‐born, with 57% and 48%, respectively, identifying as women. In terms of socio‐economic status, 19.47% (*n* = 51) of Filipino Americans identified as lower or lower middle class, 58.01% (*n* = 152) as middle class, and 22.52% (*n* = 59) as upper middle or upper class. Among Korean Americans, 35.55% (*n* = 123) identified as lower or lower middle class, 45.95% (*n* = 159) as middle class, and 18.5% (*n* = 64) as upper middle or upper class. Participants were offered $50 for completing the online survey and $40 for the paper survey participants as incentives. More information about the MLSAAF data can be found elsewhere (Choi et al., 2018).

### Measures

Unless noted otherwise, each measure was on a 5‐point Likert scale (e.g., 1 *= never* to 5 *= always*) and averaged to construct in a way that a higher score indicates a higher level of the construct. Also, all measures displayed high reliability for both Filipino Americans and Korean Americans (see Table 2).

#### General racial discrimination

A total of five items was used to ask how often the following incidents have occurred to the respondents. Three items from the MLSAAF (Choi et al., 2018) included “I have felt discriminated [against] by Whites,” “by other Asians,” or “by other racial/ethnic minorities like Blacks or Hispanics.” Two items from Phinney et al. (1998) were “my teachers or supervisors” or “classmates or co‐workers at work treat me unfairly because I am Filipino/Korean/Asian.” Responses were recorded on a 5‐point Likert scale ranging from 1 (almost never) to 5 (almost always).

#### COVID‐19‐specific racial discrimination

A total of nine items were used to measure COVID‐19‐related racial discrimination. Seven items were from the Pandemic Asian Discrimination Scale (Liu et al., 2020). For example, items included: “Someone has made a comment about Chinese/Asian people being the source of the virus” or “I have been verbally assaulted because I am Asian due to COVID‐19.” Two items were added to gauge the experiences of vicarious COVID‐19 racial discrimination (namely, “Someone close to me has been verbally assaulted because they are Asian due to COVID‐19” and “Someone close to me has been physically assaulted because they are Asian due to COVID‐19”). Each of these items was rated on a dichotomous scale, with 0 indicating no occurrence and 1 indicating affirmative experiences. By summing up the scores for each participant's responses, an aggregate score was obtained to quantify the overall extent of COVID‐19 racial discrimination experiences.

#### Fair‐society belief

Three items were utilized to assess the belief that the system in America is fair (Flanagan, Syvertsen, & Stout, 2007). Example items include “People get fair treatment in America, no matter who they are” and “In America, you have an equal chance no matter where you come from or what race or ethnicity you are.”

#### Anti‐racism action

Thirteen items from the Anti‐Racism Action Scale (Aldana et al., 2019) were used to measure how often respondents were engaged in anti‐racism actions in response to discriminatory experiences during the COVID‐19 pandemic. Consistent with Aldana et al. (2019), these 13 items were further separated into three different subscales: 5‐item interpersonal action scale (e.g., “challenged or checked a family or friend who uses a racial slur or makes a racist joke”), 3‐item communal action scale (e.g., “attended a meeting on an issue related to race, ethnicity, discrimination, and/or segregation”), and 5‐item political change action (e.g., “organized your own action project on an issue related to race, ethnicity, discrimination and/or segregation”). We used the mean score of items for each subscale as indicators of anti‐racism action on the interpersonal, communal, and political levels.

#### Nativity

Nativity was coded as 0 for foreign‐born and 1 for U.S.‐born.

#### Demographic variables

Several demographic characteristics were included in the analysis as covariates. This encompassed age, gender (with male serving as the reference category and a dummy variable employed for female and nonbinary), and self‐reported family socioeconomic status (rated on a scale of 1 for the lower class to 5 for the upper class).

### Analyses plan

First, both univariate and bivariate descriptive statistics were examined, including the calculation of means and variances for individual elements and overall constructs, confirming their reliability and assessing correlations among constructs across ethnic groups. Subsequently, we conducted path analyses within the structural equation modeling framework to test the mediating effects of fair‐society belief on the relationships between racial discrimination and anti‐racism action. General and COVID‐19‐related racial discrimination were considered independent variables, with fair‐society beliefs acting as a mediator. The dependent variables were three facets of anti‐racism action. We employed bootstrapping techniques, as outlined by Bollen and Stine (1990), to assess the mediating effects of racial discrimination on outcomes through fair‐society beliefs. Data from 265 Filipino and 347 Korean Americans were analyzed using STATA version 16.1. Furthermore, multigroup analyses were performed to evaluate if the mediating effects of fair‐society beliefs varied by nativity.

Although we had very low rates of missing data (e.g., the highest was 1.15% for Filipino Americans' SES), full information maximum likelihood (FLML) estimation was used to treat the missing cases. This approach maximized the use of available data from each case for our calculations, ensuring a robust analysis despite the minimal missing data.

## RESULTS

### Descriptive statistics

Table 1 summarizes the descriptive statistics for Filipino Americans and Korean Americans. Specifically, both Filipino American and Korean American young adults reported a moderate level of fair‐society belief. Filipino Americans reported higher levels of COVID‐19‐specific racial discrimination and all aspects of anti‐racist activism than Korean Americans. Among Filipino Americans, foreign‐born individuals reported higher levels of communal action and political change action. Among Korean Americans, U.S.‐born individuals reported higher COVID‐19 related racial discrimination but lower fair‐society belief (see Table 1).

**TABLE 1:** Descriptive statistics of the study variables across and within ethnic groups by nativity.

Variables	Total (*n* = 612)		Filipino American		Korean American		Diff. by nativity	
	Filipino Americans (*n* = 265)	Korean Americans (*n* = 347)	FA Foreign‐born (FF) (*n* = 76)	FA U.S.‐born (FU) (*n* = 189)	KA Foreign‐born (KF) (*n* = 138)	KA U.S.‐born (KU) (*n* = 209)	Within Foreign‐born (FF vs. KF)	Within U.S.‐born (FU vs. KU)
Demographic characteristics								
Nativity (U.S.‐born)	189 (71.3%)	209 (60.2%)	−	−	−	−	−	−
Gender (Woman)	153 (57.7%)	168 (48.4%)	43 (56.58%)	110 (58.20%)	71 (51.45%)	97 (46.41%)	−	−
Gender (Nonbinary)	5 (1.90%)	7 (2.00%)	3 (3.95%)	2 (1.06%)	2 (1.45%)	5 (2.39%)	−	−
Age	21.5 (1.8)*	21.2 (1.9)	21.54 (1.83)	21.55 (1.84)	21.76 (1.87)***	20.89 (1.77)		***
SES	3.0 (0.7)***	2.76 (0.8)	2.79 (0.70)	3.09 (0.75)***	2.72 (0.80)	2.78 (0.85)*		***
Predictors								
General racial discrimination	1.65 (0.6)	1.70 (0.7)	1.59 (0.62)	1.67 (0.65)	1.75 (0.74)	1.67 (0.67)	^†^	
COVID‐19 racial discrimination	2.53 (2.2)*	2.14 (1.4)	2.83 (2.50)**	2.41 (2.13)	1.86 (1.81)	2.33 (2.09)*	***	
Mediators								
Fair society belief	1.73 (.96)	1.72 (.85)	1.78 (0.94)	1.72 (0.97)	1.84 (0.96)*	1.64 (0.76)		
Outcomes								
Anti‐racist interpersonal action	3.10 (1.2)***	2.76 (1.0)	3.26 (1.20)	3.01 (1.16)	2.83 (0.99)	2.72 (1.01)	**	**
Anti‐racist communal action	1.86 (1.0)^†^	1.71 (1.0)	2.06 (1.13)*	1.78 (1.01)	1.75 (1.04)	1.68 (0.92)	*	
Anti‐racist political change action	1.69 (0.7)**	1.54 (0.7)	1.86 (0.80)**	1.62 (0.67)	1.59 (0.77)	1.5 (0.66)	*	^†^

*Note*: Mean (SD) for continuous variables or sample size (percentage) for categorical variables. Denotes *t‐test* statistical differences of the study variables between FA and KA (overall ethnic differences), Foreign‐born FA and U.S.‐born FA, Foreign‐born KA, and U.S.‐born KA, within nativity ethnic difference.

* *p* < .05.

** *p* < .01.

*** *p* < .001.

^†^ *p* < .1.

Table 2 shows the correlation among study variables. Both general discrimination and COVID‐related discrimination were positively correlated with all three types of anti‐racism action. For Filipino Americans, fair‐society belief was not correlated with both general and COVID‐related discrimination and was negatively correlated only with political anti‐racism action. For Korean Americans, fair‐society belief was negatively correlated with both general and COVID‐related discrimination and was negatively correlated with all three types of anti‐racism action.

**TABLE 2:** Mean, standard deviations, and correlation of the study variables.

Variables	1	2	3	4	5	6	7	8	9	10	11
(1) Interpersonal	–	0.40*	0.50*	−0.34*	0.32*	0.45*	0.05	0.28*	0.04	−0.1	0.01
(2) Communal	0.45*	–	0.60*	−0.18*	0.23*	0.23*	0.01	0.19*	0.09	−0.13*	0.09
(3) Political change	0.46*	0.68*	–	−0.21*	0.31*	0.33*	0.03	0.22*	0.05	−0.16*	0.11
(4) Fair society belief	−0.07	−0.07	−0.11*	–	−0.14*	−0.23*	−0.08	−0.19*	−0.04	−0.03	0.01
(5) General discrimination	0.40*	0.28*	0.32*	−0.03	–	0.43*	0.14*	0.19*	0.15*	0.07	0
(6) COVID‐specific discrimination	0.38*	0.29*	0.25*	−0.06	0.55*	–	−0.01	0.1	0.12	−0.08	−0.09
(7) Age^a^	0.04	−0.06	−0.02	−0.06	0.12*	−0.07	–	0.09	0.03	0	0.07
(8) Female^a^	0.23*	0.13*	0.17*	−0.18*	0.12*	0.08	0.03	–	−0.16*	0.02	0.09
(9) Nonbinary^a^	0.08	0.1	0.13*	−0.12*	0.07	0.06	0.01	−0.14*	–	−0.1	0
(10) Nativity^a^	−0.05	−0.04	−0.06	−0.12*	−0.06	0.11*	−0.23*	−0.05	0.03	–	0.19*
(11) Socioeconomic status^a^	−0.05	−0.05	−0.05	0.05	−0.14*	−0.11*	−0.06	−0.03	0.07	0.04	–
Alpha for Filipino Americans	0.89	0.81	0.76	0.94	0.77	0.80	N/A	N/A	N/A	N/A	N/A
Alpha for Korean Americans	0.84	0.81	0.80	0.92	0.84	0.73	N/A	N/A	N/A	N/A	N/A

*Note*: Below the diagonal are correlations for Filipino Americans, and above the diagonal are correlations for Korean Americans.

* *p* < .05.

** *p* < .01.

*** *p* < .001.

^a^ Coefficient alpha is N/A for the variables measured by a single item.

### Mediation analyses

**FIGURE 1:**
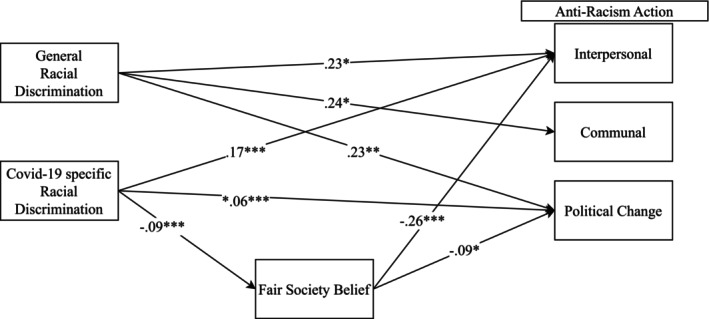
Path analysis result for Filipino Americans. *p < .05, **p < .01, ***p < .001. Several nonsignificant paths and controls for the outcome variable are not shown for simplicity.

Figure 1 shows the statistically significant paths for Filipino Americans, and the model fits the data well. The fit indices were χ^2^(19) = 43.234, *p* < .001, CFI = 0.906, and RMSEA = 0.078. While general discrimination was not significantly associated with fair‐society belief, it was positively associated with all three aspects of anti‐racism action directly (Interpersonal *b* = 0.23, *p* < .05; Communal *b* = 0.24, *p* < .05; Political change *b* = 0.23, *p* < .05). On the other hand, COVID‐19‐related discrimination was negatively associated with fair‐society belief (*b* = −0.09, *p* < .001) and positively associated with interpersonal (*b* = 0.17, *p* < .001) and political change (*b* = 0.06, *p* < .001) anti‐racism action. Fair‐society belief was negatively associated with interpersonal (*b* = −0.26, *p* < .001) and political change (*b* = −0.09, *p* < .05) aspects of anti‐racism action. Moreover, COVID‐19‐related discrimination was indirectly related to interpersonal anti‐racism action via fair‐society belief (*b* = 0.05, *p* < .01).

**FIGURE 2:**
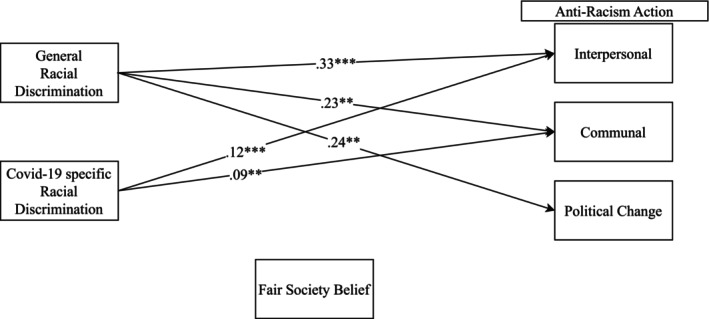
Path analysis result for Korean Americans. *p < .05, **p < .01, ***p < .001. Several nonsignificant paths and controls for the outcome variable are not shown for simplicity.

Figure 2 shows the statistically significant paths for Korean Americans. The model fit the data well. The fit indices were χ^2^(19) = 49.644, *p* < .001, CFI = 0.928, and RMSEA = 0.068. General discrimination was not significantly associated with fair‐society belief, but it was positively associated with all three aspects of anti‐racism action directly (interpersonal *b* = 0.33, *p* < .001; communal *b* = 0.23, *p* < .01; political change *b* = 0.24, *p* < .001). COVID‐19‐specific discrimination was also not significantly associated with fair‐society belief, while it was positively associated with interpersonal (*b* = 0.12, *p* < .001) and political change (*b* = 0.09, *p* < .01) anti‐racism action. Fair‐society belief was not associated with any type of anti‐racism action.

### Multigroup analysis

The findings from the multigroup SEM analysis showed that there was a difference in the model path coefficients between the U.S.‐born and the Foreign‐born. For Filipino Americans, this multigroup model was an adequate fit to the data, χ^2^(28) = 54.128, *p* < .001, CFI = 0.917, and RMSEA = 0.084. The model explained 34% and 53% of variance in anti‐racism action for U.S.‐born and foreign‐born Filipino Americans, respectively. For U.S.‐born Filipino Americans, the relationship between COVID‐19‐related discrimination and interpersonal anti‐racism action was mediated by fair‐society belief (*b* = 0.05, *p* < .01), whereas there was no significant indirect effect among foreign‐born Filipino Americans. The multigroup model also showed an adequate fit for Korean Americans. The fit indices were χ^2^(28) = 52.713, *p* < .001, CFI = 0.943, and RMSEA = 0.071. The model explained 45% and 26% of variance in anti‐racism action for U.S.‐born and foreign‐born Korean Americans, respectively. No indirect effect was shown as fair‐society belief was not associated with anti‐racism action among both groups.

### Sensitivity analysis

For sensitivity analysis, multigroup analysis by gender was also examined. However, there were no gender differences in the model path coefficient.

## DISCUSSION

The present study tested whether fair‐society belief mediates the relationships between racial discrimination and anti‐racist action among Filipino American and Korean American young adults and how these mediating relationships further vary by nativity status. The findings of this study suggest that Asian Americans actively make their voices heard through anti‐racist actions in response to racial discrimination. These results refute the “model minority” stereotypes that Asian Americans are conformist, apolitical, and civically unengaged (Wray‐Lake et al., 2017). Rather, Asian American young adults, particularly those who have experienced racial discrimination, are involved in anti‐racist action on multiple levels. For some Asian ethnic groups, experiences of racial discrimination affect their perceptions of fairness in American society, which in turn motivates anti‐racist actions aimed at achieving racial justice. This result is consistent with findings from extant literature that fair‐society beliefs predict civic values and behaviors (Flanagan, 2012; Flanagan, Cumsille, et al., 2007). Yet, the present study further documents that fair‐society belief is only predictive of anti‐racist action for some Asian ethnic groups, not all.

Specifically, the proposed mediating role of fair‐society beliefs was supported among U.S.‐born Filipino young adults, indicating that the COVID‐19‐specific racial discrimination was associated with lower fair‐society beliefs. Lower fair‐society beliefs, in turn, were related to more anti‐racist action. Previous studies supported the significance of fair‐society beliefs (Wray‐Lake et al., 2015) and related constructs (e.g., critical reflection; Golden & Byrd, 2022) as a mediator between minority youth's experiences and critical civic action. However, our findings contrast with Wray‐Lake et al. (2015), who found positive relationships between fair‐society beliefs and community engagement among Asian American youth. One possible reason for this difference may be that they focused on community engagement that is not particularly related to race, whereas we studied anti‐racist activism during the pandemic. Low fair‐society belief in the context of racial discrimination experiences and widespread anti‐Asian hate during the pandemic may portray one's critical reflection and frustration toward racism, which motivate acts of resistance to change oppressive conditions (Golden & Byrd, 2022; Quiles et al., 2023). The findings of this study highlight the importance of promoting access to opportunities for anti‐racist action, targeted towards Filipino young adults, that can channel their discrimination experiences and fair‐society beliefs into efforts to fight against racism. By including content about race and systemic racism, institutions for higher education and community organizations can help Filipino young adults make structural contributions to racism and inequity, rather than internalizing or denying racism, and stimulate their anti‐racist action. Relatedly, the findings raise a concern to the calls to reduce dialogues about racism and to promote colorblindness, in that such initiatives can limit young adults' capacities to critically reflect on social structures and make contributions to social justice (Golden & Byrd, 2022).

The study findings also revealed that fair‐society belief only mediated the relationship between COVID‐19‐specific discrimination and interpersonal anti‐racist action, but not communal or political actions. This pattern likely emerges from the distinctive characteristics of anti‐Asian discrimination during the COVID‐19 pandemic, which predominantly occurred through direct personal encounters rather than institutional channels (Yellow Horse et al., 2022). The measurement of COVID‐19 discrimination (Liu et al., 2020) used in this study specifically captured immediate face‐to‐face incidents, such as direct verbal confrontations or expressions of suspicion, which may correspondingly elicit interpersonal‐level responses. When young adults experience discrimination in immediate social interactions, they would be more likely to respond through direct interpersonal interventions rather than broader organizational or political engagement. Nevertheless, among U.S.‐born Filipino Americans, we found that decreased fair‐society beliefs were also linked to increased political anti‐racist action, suggesting that personal beliefs of unfairness can also promote wider social justice engagement.

It is also important to note that the mediating role of fair‐society belief was only identified among U.S.‐born Filipino Americans, but not among Korean Americans or foreign‐born Filipino Americans. Filipino Americans, particularly those born in the United States, generally demonstrate higher levels of acculturation into American society compared to Korean Americans (Min, 2006; Vigdor, 2008). This greater sense of integration into American society may make experiences of discrimination particularly impactful on their beliefs about societal fairness. When U.S.‐born Filipino Americans, who have been raised to believe that American society is fair, encounter discrimination that challenges these beliefs, they may be more likely to engage in anti‐racist action as a way to address this cognitive dissonance. This finding aligns with previous research suggesting that greater societal integration can lead to heightened awareness of and responses to discrimination (Kibria, 2000).

The absence of the mediation effect of fair‐society belief in Korean Americans and foreign‐born Filipino Americans suggests that these groups may process and respond to discrimination through different mechanisms. Korean Americans often maintain distinct cultural spaces and social networks (Min, 2006), which could influence how they interpret discriminatory experiences in relation to broader American society. These findings highlight how both ethnic background and immigration history interact to shape the pathways between discrimination experiences and anti‐racist engagement.

One of the limitations of this study was the use of cross‐sectional data. For example, it is possible that those who believe society is fair may be less likely to categorize negative experiences as discriminatory, and instead may attribute these experiences to individual deficit or circumstances. Thus, while experiences of discrimination may jeopardize beliefs in a fair society, a maintenance of such belief may conversely impact minority individuals' own perception and experience of such discrimination. Future longitudinal studies can examine these dynamics between the variables. Another limitation is that this study focused on the experiences of two Asian ethnic groups in the Midwestern region in the United States, limiting the generalizability of the findings to other Asian ethnic groups and regions. However, given a dearth of studies on Asian Americans in the Midwest, this study expands literature on limited studies on young Asian Americans.

## CONCLUSION

This study advances our understanding of how racial discrimination shapes anti‐racist action among Asian American young adults through their beliefs about societal fairness. First, we found that discrimination can mobilize Asian Americans to engage in anti‐racist actions, challenging the stereotype of Asian Americans being politically passive. Second, fair‐society belief may serve as an important mediating factor in the link between COVID‐19‐specific discrimination and interpersonal anti‐racist action, particularly for U.S.‐born Filipino Americans. This finding suggests that when discrimination challenges one's belief in societal fairness, it can motivate individuals to take action against racial injustice. The study also highlights important variations in how discrimination experiences are processed and acted upon across Asian American subgroups and nativity backgrounds. Notably, the mediating role of fair‐society belief was unique to U.S.‐born Filipino Americans, suggesting that the pathways from discrimination experiences to anti‐racist action are shaped by complex intersections of ethnicity and immigration history. The current study findings caution against treating Asian Americans as a monolithic group and underscore the importance of considering specific ethnic and generational contexts when developing intervention programs to promote anti‐racist action among Asian American young adults.

## FUNDING INFORMATION

This work was supported by the grants from the Eunice Kennedy Shriver National Institute of Child Health & Human Development, R01HD073200 (PI: Fourth author) and Russell Sage Foundation, 2005‐24450 (PI: Fourth author).

## CONFLICT OF INTEREST STATEMENT

The authors report no conflict of interests.

## INFORMED CONSENT

All study participants received thorough informed consent and assent process.

## Data Availability

The data sets analyzed in the current study are not publicly available but can be available from the fourth author if certain conditions are met.

## References

[jora70023-bib-0001] Aldana, A. , Bañales, J. , & Richards‐Schuster, K. (2019). Youth anti‐racist engagement: Conceptualization, development, and validation of an anti‐racism action scale. Adolescent Research Review, 4(4), 369–381. 10.1007/s40894-019-00113-1

[jora70023-bib-0002] Armenta, B. E. , Lee, R. M. , Pituc, S. T. , Jung, K. R. , Park, I. J. , Soto, J. A. , & Schwartz, S. J. (2013). Where are you from? A validation of the foreigner objectification scale and the psychological correlates of foreigner objectification among Asian Americans and Latinos. Cultural Diversity and Ethnic Minority Psychology, 19(2), 131–142. 10.1037/a0031547 23647327 PMC7869143

[jora70023-bib-0003] Ballard, P. J. (2015). Longitudinal links between discrimination and civic development among Latino and Asian adolescents. Journal of Research on Adolescence, 26(4), 723–737. 28453198 10.1111/jora.12221

[jora70023-bib-0004] Bañales, J. , Mathews, C. , Hayat, N. , Anyiwo, N. , & Diemer, M. A. (2020). Latinx and black young adults' pathways to civic/political engagement. Cultural Diversity and Ethnic Minority Psychology, 26(2), 176–188. 10.1037/cdp0000271 30816755

[jora70023-bib-0005] Berman, G. , & Paradies, Y. (2010). Racism, disadvantage and multiculturalism: Towards effective anti‐racist praxis. Ethnic and Racial Studies, 33(2), 214–232. 10.1080/01419870802302272

[jora70023-bib-0058] Bollen, K. A. , & Stine, R. (1990). Direct and Indirect Effects: Classical and Bootstrap Estimates of Variability. Sociological Methodology, 20, 115–140. 10.2307/271084

[jora70023-bib-0006] Cheah, C. S. , Wang, C. , Ren, H. , Zong, X. , Cho, H. S. , & Xue, X. (2020). COVID‐19 racism and mental health in Chinese American families. Pediatrics, 146(5), e2020021816. 10.1542/peds.2020-021816 32873719

[jora70023-bib-0007] Choi, Y. , Park, M. , Lee, J. P. , Yasui, M. , & Kim, T. Y. (2018). Explicating acculturation strategies among Asian American youth: Subtypes and correlates across Filipino and Korean Americans. Journal of Youth and Adolescence, 47(10), 2181–2205. 10.1007/s10964-018-0862-1 29881910 PMC6286232

[jora70023-bib-0008] Choi, Y. , Park, M. , Noh, S. , Lee, J. P. , & Takeuchi, D. T. (2020). Asian American mental health: Longitudinal trend and explanatory factors among Filipino and Korean Americans. Social Science and Medicine: Population Health, 10, 100542. 10.1016/j.ssmph.2020.100542 PMC6994703 32021900

[jora70023-bib-0009] Christophe, N. K. , Martin Romero, M. Y. , Hope, E. , & Stein, G. L. (2022). Critical civic engagement in black college students: Interplay between discrimination, centrality, and preparation for bias. American Journal of Orthopsychiatry, 92(2), 144–153. 10.1037/ort0000600 34941293

[jora70023-bib-0010] Coll, C. G. , Crnic, K. , Lamberty, G. , Wasik, B. H. , Jenkins, R. , Garcia, H. V. , & McAdoo, H. P. (1996). An integrative model for the study of developmental competencies in minority children. Child Development, 67(5), 1891–1914. 10.2307/1131600 9022222

[jora70023-bib-0011] Cooc, N. , & Kim, G. M. (2021). The roles of racial discrimination and English in civic outcomes for Asian Americans and Pacific islanders. Cultural Diversity and Ethnic Minority Psychology, 27(3), 483–494. 10.1037/cdp0000443 33719470

[jora70023-bib-0012] Correia, M. E. (2010). Determinants of attitudes toward police of Latino immigrants and non‐immigrants. Journal of Criminal Justice, 38(1), 99–107. 10.1016/j.jcrimjus.2009.11.012

[jora70023-bib-0013] Darling‐Hammond, S. , Michaels, E. K. , Allen, A. M. , Chae, D. H. , Thomas, M. D. , Nguyen, T. T. , Mujahid, M. M. , & Johnson, R. C. (2020). After “the China virus” went viral: Racially charged coronavirus coverage and trends in bias against Asian Americans. Health Education and Behavior, 47(6), 870–879. 10.1177/1090198120957949 32911985 PMC7488172

[jora70023-bib-0014] Deaux, K. , Reid, A. , Martin, D. , & Bikmen, N. (2006). Ideologies of diversity and inequality: Predicting collective action in groups varying in ethnicity and immigrant status. Political Psychology, 27(1), 123–146.

[jora70023-bib-0015] Diemer, M. A. , & Rapa, L. J. (2016). Unraveling the complexity of critical consciousness, political efficacy, and political action among marginalized adolescents. Child Development, 87(1), 221–238. 10.1111/cdev.12446 26505744

[jora70023-bib-0016] Flanagan, C. A. (2012). Teenage citizens: The political theories of the young. Harvard University Press.

[jora70023-bib-0017] Flanagan, C. A. , Cumsille, P. , Gill, S. , & Gallay, L. S. (2007). School and community climates and civic commitments: Patterns for ethnic minority and majority students. Journal of Educational Psychology, 99(2), 421–431.

[jora70023-bib-0018] Flanagan, C. A. , Syvertsen, A. K. , Gill, S. , Gallay, L. S. , & Cumsille, P. (2009). Ethnic awareness, prejudice, and civic commitments in four ethnic groups of American adolescents. Journal of Youth and Adolescence, 38(4), 500. 10.1007/s10964-009-9394-z 19636724

[jora70023-bib-0019] Flanagan, C. A. , Syvertsen, A. K. , & Stout, M. D. (2007). Civic measurement models: Tapping adolescents' civic engagement. CIRCLE Working Paper 55. Center for Information and Research on Civic Learning and Engagement (CIRCLE).

[jora70023-bib-0020] Gee, G. C. , Ro, A. , Shariff‐Marco, S. , & Chae, D. (2009). Racial discrimination and health among Asian Americans: Evidence, assessment, and directions for future research. Epidemiologic Reviews, 31(1), 130–151. 10.1093/epirev/mxp009 19805401 PMC4933297

[jora70023-bib-0021] Golden, A. R. , & Byrd, C. M. (2022). Examining critical reflection as a mediator between school racial climate experiences and anti‐racist action. Journal of Research on Adolescence, 32(3), 1109–1119. 35709012 10.1111/jora.12778 PMC9542284

[jora70023-bib-0022] Ho, I. K. , & Çabuk, K. (2023). The impact of racial discrimination on the health of Asian Americans during the COVID‐19 pandemic: A scoping review. Ethnicity & Health, 28(7), 957–982. 37160688 10.1080/13557858.2023.2208312

[jora70023-bib-0023] Hope, E. C. , Hoggard, L. S. , & Thomas, A. (2015). Emerging into adulthood in the face of racial discrimination: Physiological, psychological, and sociopolitical consequences for african american youth. Translational Issues in Psychological Science, 1(4), 342–351. 10.1037/tps0000041

[jora70023-bib-0024] Hope, E. C. , & Jagers, R. J. (2014). The role of sociopolitical attitudes and civic education in the civic engagement of black youth. Journal of Research on Adolescence, 24(3), 460–470. 10.1111/jora.12117

[jora70023-bib-0025] Hope, E. C. , Volpe, V. V. , Briggs, A. S. , & Benson, G. P. (2022). Anti‐racism activism among black adolescents and emerging adults: Understanding the roles of racism and anticipatory racism‐related stress. Child Development, 93(3), 717–731. 10.1111/cdev.13744 35211959 PMC9306571

[jora70023-bib-0026] Iwamoto, D. K. , Kane, J. C. , Negi, N. J. , Collado, A. , & Tofighi, D. (2022). Racial discrimination, distress, coping motives, and alcohol‐related problems among US‐born Asian American young adults. Asian American Journal of Psychology, 13(2), 177.

[jora70023-bib-0027] Jensen, L. A. (2010). Immigrant youth in the United States: Coming of age among diverse civic cultures.

[jora70023-bib-0028] Jun, J. , Kim, J. K. , & Woo, B. (2021). Fight the virus and fight the bias: Asian Americans' engagement in activism to combat Anti‐Asian COVID‐19 racism. Race and Justice, 14(2), 21533687211054165. 10.1177/21533687211054165

[jora70023-bib-0029] Junn, J. (2007). From coolie to model minority: US immigration policy and the construction of racial identity. Du Bois Review: Social Science Research on Race, 4(2), 355–373.

[jora70023-bib-0030] Kao, G. , & Tienda, M. (2022). Optimism and achievement: The educational performance of immigrant youth. In The new immigrants and American schools (pp. 83–101). Routledge.

[jora70023-bib-0031] Kibria, N. (2000). Race, ethnic options, and ethnic binds: Identity negotiations of second‐generation Chinese and Korean Americans. Sociological Perspectives, 43(1), 77–95. 10.2307/1389783

[jora70023-bib-0032] Liu, C. H. , Zhang, E. , & Hahm, H. C. (2020). COVID‐19‐related discrimination scale.

[jora70023-bib-0033] Lui, P. P. , Parikh, K. , Katedia, S. , & Jouriles, E. N. (2022). Anti‐Asian discrimination and antiracist bystander behaviors amid the COVID‐19 outbreak. Asian American Journal of Psychology, 13(3), 295–304. 10.1037/aap0000258

[jora70023-bib-0034] Matriano, R. , Atkin, A. L. , Yoo, H. C. , & Gabriel, A. K. (2021). Asian American college students’ support for black lives matter: Role of internalizing the model minority myth and critical reflection. Asian American Journal of Psychology, 12(4), 291–300. 10.1037/aap0000250

[jora70023-bib-0035] Mattis, J. S. , Hearn, K. D. , & Jagers, R. J. (2002). Factors predicting communal attitudes among African American men. Journal of Black Psychology, 28(3), 197–214. 10.1177/0095798402028003002

[jora70023-bib-0036] Menjívar, C. , & Bejarano, C. (2004). Latino immigrants' perceptions of crime and police authorities in the United States: A case study from the Phoenix metropolitan area. Ethnic and Racial Studies, 27(1), 120–148.

[jora70023-bib-0037] Min, P. G. (2006). Asian Americans: Contemporary trends and issues. Pine Forge Press.

[jora70023-bib-0038] Park, M. , Woo, B. , Jung, H.‐M. , Jeong, E. , Choi, Y. , Takeuchi, D. , & Peregrina, H. N. (2024). COVID‐19, racial discrimination and civic engagement among Filipino American and Korean American young adults. Emerging Adulthood, 12(2), 236–251.

[jora70023-bib-0039] Perreira, K. M. , Harris, K. M. , & Lee, D. (2006). Making it in America: High school completion by immigrant and native youth. Demography, 43(3), 511–536. 17051825 10.1353/dem.2006.0026

[jora70023-bib-0040] Phinney, J. S. , Madden, T. , & Santos, L. J. (1998). Psychological variables as predictors of perceived ethnic discrimination among minority and immigrant adolescents. Journal of Applied Social Psychology, 28(11), 937–953.

[jora70023-bib-0041] Quiles, T. B. , Hoyt, L. T. , Dotson, M. P. , Castro, E. M. , May, M. , & Cohen, A. K. (2023). Who has to act? A qualitative exploration of emerging adults' critical consciousness during the COVID‐19 pandemic. American Journal of Community Psychology, 71(1–2), 136–146. 36594881 10.1002/ajcp.12638

[jora70023-bib-0042] Rumbaut, R. G. (2008). Reaping what you sow: Immigration, youth, and reactive ethnicity. Applied Development Science, 12(2), 108–111. 10.1080/10888690801997341

[jora70023-bib-0043] Saavedra, J. A. , & Yoo, H. C. (2023). Translating critical reflection into collective action: The mediating role of Asian American racial identity ideological values. American Journal of Community Psychology, 72(1–2), 60–74. 10.1002/ajcp.12681 37200215

[jora70023-bib-0044] Sánchez‐Jankowski, M. (2002). Minority youth and civic engagement: The impact of group relations. Applied Developmental Science, 6(4), 237–245. 10.1207/S1532480XADS0604_11

[jora70023-bib-0045] Seaton, E. K. , Gee, G. C. , Neblett, E. , & Spanierman, L. (2018). New directions for racial discrimination research as inspired by the integrative model. American Psychologist, 73(6), 768–780. 10.1037/amp0000315 30188165

[jora70023-bib-0046] Vigdor, J. L. (2008). Measuring immigrant assimilation in the United States (Civic Report No. 53): Vol. d. c. Manhattan Institute. https://media4.manhattan‐institute.org/pdf/cr_53.pdf

[jora70023-bib-0047] Watts, R. J. , Diemer, M. A. , & Voight, A. M. (2011). Critical consciousness: Current status and future directions. New Directions for Child and Adolescent Development, 2011(134), 43–57. 22147600 10.1002/cd.310

[jora70023-bib-0048] Watts, R. J. , Williams, N. C. , & Jagers, R. J. (2003). Sociopolitical development. American Journal of Community Psychology, 31(1–2), 185–194. 10.1023/a:1023091024140 12741699

[jora70023-bib-0049] White‐Johnson, R. L. (2012). Prosocial involvement among African American young adults: Considering racial discrimination and racial identity. Journal of Black Psychology, 38(3), 313–341. 10.1177/0095798411420429

[jora70023-bib-0050] Wong, J. S. , Ramakrishnan, S. K. , Lee, T. , Junn, J. , & Wong, J. (2011). Asian American political participation: Emerging constituents and their political identities. Russell Sage Foundation.

[jora70023-bib-0051] Wray‐Lake, L. , Rote, W. M. , Gupta, T. , Godfrey, E. , & Sirin, S. (2015). Examining correlates of civic engagement among immigrant adolescents in the United States. Research in Human Development, 12(1–2), 10–27.

[jora70023-bib-0052] Wray‐Lake, L. , Syvertsen, A. K. , & Flanagan, C. A. (2008). Contested citizenship and social exclusion: Adolescent Arab American immigrants' views of the social contract. Applied Development Science, 12(2), 84–92.

[jora70023-bib-0053] Wray‐Lake, L. , Tang, J. , & Victorino, C. (2017). Are they political? Examining Asian American college students' civic engagement. Asian American Journal of Psychology, 8(1), 31–42. 10.1037/aap0000061

[jora70023-bib-0054] Wray‐Lake, L. , Wells, R. , Alvis, L. , Delgado, S. , Syvertsen, A. K. , & Metzger, A. (2018). Being a Latinx adolescent under a trump presidency: Analysis of Latinx youth's reactions to immigration politics. Children and Youth Services Review, 87, 192–204.

[jora70023-bib-0055] Yellow Horse, A. J. , Jeung, R. , & Matriano, R. (2022). Stop AAPI Hate National Report 3/19/20–12/31/21. Stop AAPI Hate.

[jora70023-bib-0056] Zhang, Y. , Zhang, L. , & Benton, F. (2022). Hate crimes against Asian Americans. American Journal of Criminal Justice, 47(3), 441–461. 10.1007/s12103-020-09602-9 33437139 PMC7790522

[jora70023-bib-0057] Zhou, L. (2022). The Stop Asian Hate movement is at a crossroads. Vox. https://www.vox.com/22820364/stop‐asian‐hate‐movement‐atlanta‐shootings

